# Clinical characteristics of apixaban prescription in AF patients with single dose-reduction criterion: the ASPIRE (efficAcy and safety of aPixaban in rEal-world practice in Korean frail patients with atrial fibrillation) study

**DOI:** 10.3389/fcvm.2024.1367623

**Published:** 2024-06-10

**Authors:** JungMin Choi, So-Ryoung Lee, Soonil Kwon, Hyo-Jeong Ahn, Kyung-Yeon Lee, Jong-Sung Park, Jong-Il Choi, Sung Ho Lee, Jung Ho Heo, Il-Young Oh, Young Keun On, Hee Tae Yu, Kwang-No Lee, Nam-Ho Kim, Hyung Wook Park, Ki Hong Lee, Seung Yong Shin, Seil Oh, Gregory Y. H. Lip, Seongwook Han, Eue-Keun Choi

**Affiliations:** ^1^ Department of Internal Medicine, Seoul National University Hospital, Seoul, Republic of Korea; ^2^ Department of Internal Medicine, Seoul National University College of Medicine, Seoul, Republic of Korea; ^3^ Department of Internal Medicine, Seoul Metropolitan Government-Seoul National University Boramae Medical Center, Seoul, Republic of Korea; ^4^ Department of Cardiology, Dong-A University Hospital, Busan, Republic of Korea; ^5^ Division of Cardiology, Department of Internal Medicine, Korea University College of Medicine and Korea University Anam Hospital, Seoul, Republic of Korea; ^6^ Division of Cardiology, Department of Internal Medicine, Kangbuk Samsung Hospital, Sungkyunkwan University School of Medicine, Seoul, Republic of Korea; ^7^ Department of Internal Medicine, Kosin University Gospel Hospital, Busan, Republic of Korea; ^8^ Department of Internal Medicine, Seoul National University Bundang Hospital, Gyeonggi, Republic of Korea; ^9^ Department of Cardiology, Heart Vascular Stroke Institute, Samsung Medical Center, Sungkyunkwan University School of Medicine, Seoul, Republic of Korea; ^10^ Department of Internal Medicine, Severance Cardiovascular Hospital, Yonsei University College of Medicine, Seoul, Republic of Korea; ^11^ Department of Cardiology, Ajou University School of Medicine, Suwon, Republic of Korea; ^12^ Department of Internal Medicine, Wonkwang University Hospital, Iksan, Republic of Korea; ^13^ Department of Internal Medicine, Chonnam National University Hospital, Gwangju, Republic of Korea; ^14^ Department of Cardiovascular Medicine, Chonnam National University Medical School, Gwangju, Republic of Korea; ^15^ Cardiovascular & Arrhythmia Center, Chung-Ang University Hospital, Seoul, Republic of Korea; ^16^ Liverpool Centre for Cardiovascular Science at University of Liverpool, Liverpool John Moores University and Liverpool Chest and Heart Hospital, Liverpool, United Kingdom; ^17^ Department of Clinical Medicine, Aalborg University, Aalborg, Denmark; ^18^ Division of Cardiology, Department of Internal Medicine, Keimyung University Dongsan Hospital, Daegu, Republic of Korea

**Keywords:** apixaban, atrial fibrillation, dose, clinical characteristics, off-label reduced dose

## Abstract

**Background:**

Data on off-label reduced dose risk among patients with atrial fibrillation (AF) who qualify for a single-dose reduction of apixaban is scarce.

**Objectives:**

We prospectively assessed apixaban dosing and clinical characteristics in AF patients meeting a dose reduction criterion.

**Methods:**

The multicentre, prospective cohort study, the efficAcy and Safety of aPixaban In REal-world practice in Korean frail patients with AF (ASPIRE), encompasses patients with AF who met the criteria for a single-dose reduction of apixaban and were given varying doses of apixaban, either the on-label standard dose or the off-label reduced dose.

**Results:**

Of 2,000 patients (mean age 74.3 ± 7.9 years, 55.8% women), 29.7% were ≥80 years, 62.6% weighed ≤60 kg, and 7.8% had serum creatinine ≥1.5 mg/dL. Of these, 51.3% were prescribed an off-label reduced dose of apixaban. The off-label group was characterized with older age, more comorbidities, and antiplatelet agents, while the on-label group had more prior strokes. Physicians preferred off-label reduced dose in the “marginal zone,” defined as age 75–80 years, weight 60–65 kg, and creatinine levels 1.2–1.5 mg/dL.

**Conclusions:**

In real-world clinical setting of the Korean population, off-label reduced dose apixaban was administered to nearly half of the patients who qualified for a single dose reduction. This reduced dosage was more commonly prescribed to patients with frail characteristics, while patients with a history of stroke were more often given the standard dose as per the label. A future study is planned to contrast the safety and effectiveness of the standard dose against the reduced dose of apixaban in this population.

## Introduction

Prescription of oral anticoagulation (OAC) is a crucial measure for preventing stroke in patients with atrial fibrillation (AF) (1–3). Direct oral anticoagulant (DOAC) is prioritized except for low stroke risk or contraindications (1–3). In South Korea, similar to global trends, DOAC prescriptions for patients with AF have increased rapidly over the past decade (4).

Guidelines emphasize DOAC dose reduction based on approved criteria for optimal patient benefit (1, 3, 5). However, off-label DOAC dosing, especially underdosing in Asian patients, is common (6–11). DOAC off-label underdosing is linked to a higher risk of ischemic stroke according to several (largely retrospective) observational studies (8, 11, 12).

Based on the pivotal randomised clinical trial (RCT) and practical guideline (5, 13), apixaban dose should be reduced from 5 mg twice daily to 2.5 mg twice daily in patients who met two or more criteria (age ≥80 years, body weight ≤60 kg, and serum creatinine ≥1.5 mg/dL). Given the stringent requirements for apixaban dose reduction, only 4.7% (*n* = 428) of the apixaban group was prescribed a reduced dose in the ARISTOTLE trial (13). In South Korea, apixaban 2.5 mg is widely prescribed in real-world settings and is mostly used for off-label underdosing (9, 14). The factors associated with off-label underdosing often align with traits of frail patients and dose reduction criteria (e.g., old age, underweight, and renal impairment) (9). Among anticoagulated patients with AF, older adults, those who are underweight or have renal impairment are at high risk of bleeding and stroke (15–17). Although off-label reduced dose apixaban is generally associated with a higher risk of stroke, based on previous observational studies (8, 11, 18), the risk of off-label reduced dose apixaban in patients with AF who meet a single criterion for dose reduction has not been demonstrated. E

In this study, we aimed to describe real-world apixaban dosing patterns in patients meeting a single criterion for dose reduction and assess factors related to off-label reduced dose prescriptions.

## Materials and methods

The efficacy and Safety of aPixaban In REal-world practice in Korean frail patients with atrial fibrillation (ASPIRE) study was a prospective, multicentre, non-interventional observational study covering all geographical regions of the Republic of Korea. The study enrolled participants aged >19 years with non-valvular AF receiving apixaban in the outpatient clinics of 32 centres.

The ASPIRE study aimed to delineate the effectiveness and safety results among the participating patients. The data were recorded in a common electronic database at each centre with regular audits. The data gathered was recorded in the iCReaT (Internet-based Clinical Research and Trial Management System), a web-based system for managing clinical research, which is a service provided by the Korean government. The participants were followed up on regularly at 3-month intervals via personal interviews.

Each centre's ethics committee gave their approval for the protocols, which were carried out in compliance with the principles set forth in the Declaration of Helsinki (H-2108-110-1245). This study was registered at ClinicalTrials.gov (NCT05773222). All the patients provided informed consent for inclusion in the study.

### Study population

**Figure 1:**
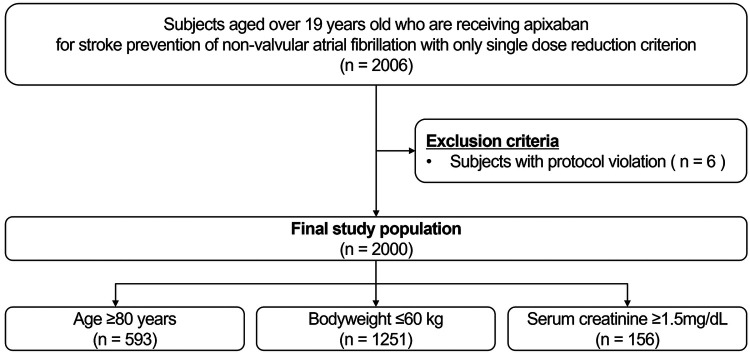
Study flow. The flow diagram of the present study is shown.

Participants aged >19 years receiving apixaban for stroke prevention due to nonvalvular AF and those with a single criterion for dose reduction for apixaban were screened. The apixaban dose reduction criteria were as follows: (1) age ≥80 years (2) body weight ≤60 kg (3) serum creatinine ≥1.5 mg/dL (13). After excluding participants with protocol violations (*n* = 6), a total of 2,000 participants were eligible for the study (Figure 1). Among the participants who met the inclusion criteria, the selection of a specific apixaban dose between 5 mg twice daily and 2.5 mg twice daily was left to the physicians. The exclusion criteria were as follows: (1) vulnerability (according to the Korean Good Clinical Practice definition) or disagreement with the study; (2) patients who had a history of clinical events, defined as primary and secondary outcomes of the study, prior to study registration after taking apixaban; and (3) satisfaction of two or more dose reduction criteria for apixaban.

### Covariates

Demographic information and anthropometric measurements including age, sex, weight, height, and body mass index were collected. Systolic and diastolic blood pressure and heart rate were also collected. Comorbidities, such as hypertension, diabetes mellitus, heart failure, history of stroke/transient ischaemic attack (TIA), bleeding, chronic kidney disease (CKD), liver disease, and malignancy were included as baseline variables. The laboratory results were as follows: complete blood count (haemoglobin and platelets), prothrombin time, international normalised ratio (INR), and chemistry (creatinine, creatinine clearance and estimated glomerular filtration rate). The CHA_2_DS_2_-VASc (score and each component: chronic heart failure, hypertension, age ≥75 years, diabetes mellitus, stroke/TIA, vascular disease history, age between 65 and 74 years, female sex), and HAS-BLED scores (score and each component: hypertension, abnormal renal and liver function, stroke history, bleeding, labile INR, age ≥65 years, drugs, or alcohol) were calculated based on the participants' comorbidities and laboratory data (19, 20). The AF diagnosis was collected according to the type [paroxysmal, non-paroxysmal (persistent, long-standing persistent, permanent), and not determined], European Heart Rhythm Association (EHRA) symptom classification, and the presence of rhythm control (21). The baseline pharmacological treatment data included OAC history, specific types of OAC, and antiplatelet therapy (APT).

### Statistical analysis

Among the baseline characteristics, continuous variables are displayed as mean ± standard deviation while categorical variables are represented by counts and corresponding percentages. Comparisons between groups were made using the Mann–Whitney U, chi-square, Kruskal–Wallis, and Fisher's exact tests.

To assess the effect of the other two components of dose reduction after one inclusion criteria has been fulfilled, we planned additional analysis on the “marginal zone”. The marginal zone was defined as an age between 75 and 80 years, weight between 60 and 65 kg, and creatinine level between 1.2–1.5 mg/dL. Among the off-label reduced group, those with at least one marginal zone value were grouped into the marginal off-label reduced group and those who did not, were grouped into the non-marginal off-label reduced group.

Factors linked with off-label reduced dose were assessed using logistic regression. For univariable logistic regression, we included marginal zone (age 75–79 years, bodyweight 60–65 kg, serum creatinine 1.2–1.5 mg/dL), sex (female), hypertension, previous history of stroke/TIA, previous history of bleeding, concomitant APT, and anaemia (22). For the multivariable logistic regression, only variables with a significant association in the univariable logistic regression were included. Specifically, in the ≥80 years group, serum creatinine levels of 1.2–1.5 mg/dL and female gender were used. In the bodyweight ≤60 kg group, the variables included were age 75–79 years, serum creatinine levels of 1.2–1.5 mg/dL, previous stroke/TIA, previous bleeding, antiplatelet use, and anaemia. Lastly, in the serum creatinine ≥1.5 mg/dL group, body weight 60–65 kg and female gender were included. Statistical significance was set at *p* < 0.05. All statistical analyses were performed using SPSS version 25 (IBM Corp., Armonk, NY, USA).

## Results

From 12 March 2020 to 15 September 2022 2,000 patients were included in this analysis. The mean age of the total population was 74.3 ± 7.9 years (55.8% women; *n* = 1,115) and the mean body weight was 60.1 ± 10.0 kg. Overall, 593 (29.7%) patients were aged ≥80 years, 1,251 (62.6%) had body weight ≤60 kg, and 156 (7.8%) had serum creatinine ≥1.5 mg/dL. Baseline characteristics of the study population according to apixaban dosage are shown in Table 1. Patients taking off-label reduced dose apixaban were more likely to have hypertension, heart failure, prior history of bleeding, CKD, and anaemia than those taking on-label standard-dose apixaban. The on-label standard-dose group was more likely to have a history of stroke or TIA. The prevalence of diabetes mellitus, liver disease, and malignancy were similar between the two groups. Concomitant APT use was more common in the off-label reduced dose group. Among the total population, 26.4% were newly prescribed OAC upon enrolment in this study, and 73.6% of participants were prescribed OAC before enrolment. Both groups had similar AF types, mostly paroxysmal (52.2%, *n* = 1,044), then persistent (35.3%, *n* = 705). The most common EHRA symptom category was IIa (39.5%, *n* = 790), followed by category I (23.9%, *n* = 478).

**Table 1:** Baseline characteristics of the whole study population according to apixaban dose.

	Total (*n* = 2,000)	Apixaban dose		*p-*value
		On–label standard–dose (*n* = 974)	Off–label reduced dose (*n* = 1,026)	
Single dose reduction criteria				
Age ≥80 years	593 (29.7)	247 (25.4)	346 (33.7)	<0.001
Body weight ≤60 kg	1,251 (62.6)	675 (69.3)	576 (56.1)	<0.001
Creatinine ≥1.5 mg/dL	156 (7.8)	52 (5.3)	104 (10.1)	<0.001
Age, years	74.3 ± 7.9	72.7 ± 7.9	75.4 ± 7.9	<0.001
<65	225 (11.3)	152 (15.6)	73 (7.1)	<0.001
65–74	691 (34.5)	410 (42.1)	281 (27.4)	<0.001
75–79	491 (24.6)	165 (16.9)	326 (31.8)	<0.001
Sex (female)	1,115 (55.8)	551 (56.6)	564 (55.0)	0.471
Bodyweight (kg)	60.1 ± 10.0	59.1 ± 9.2	60.0 ± 10.1	0.084
≤50	253 (12.7)	107 (11.0)	146 (14.2)	0.031
51–60	998 (49.9)	568 (58.3)	430 (41.9)	<0.001
>60	749 (37.5)	299 (30.7)	450 (43.9)	<0.001
CHA₂DS₂–VASc score	3.4 ± 1.2	3.1 ± 1.3	3.4 ± 1.2	<0.001
≥3	1,492 (74.6)	668 (68.6)	724 (80.3)	<0.001
HAS–BLED score*	1.6 ± 0.9	1.8 ± 1.0	1.9 ± 1.0	0.010
≥3	265 (13.7)	120 (12.6)	145 (14.8)	0.162
Comorbidities				
Hypertension	1,392 (69.9)	653 (67.0)	739 (72.0)	0.015
Diabetes Mellitus	610 (30.5)	283 (29.1)	327 (31.9)	0.172
Heart failure	476 (23.8)	207 (21.3)	269 (26.2)	0.009
Prior stroke/TIA	212 (10.6)	128 (13.1)	84 (8.2)	<0.001
Prior bleeding	143 (7.1)	54 (5.5)	89 (8.7)	0.007
CKD	218 (10.9)	66 (6.8)	152 (14.8)	<0.001
Not on dialysis	178 (8.9)	56 (5.7)	122 (11.9)	0.474
On dialysis	25 (1.3)	8 (0.8)	17 (1.7)	0.855
Previous kidney transplantation	14 (0.7)	2 (0.2)	12 (1.2)	0.175
Liver disease	77 (3.9)	35 (2.6)	42 (4.1)	0.561
Malignancy	270 (13.5)	122 (12.5)	148 (14.4)	0.214
Antiplatelet use**	125 (6.4)	43 (4.5)	82 (8.3)	0.001
SAPT	106 (5.3)	36 (3.7)	70 (6.8)	0.808
DAPT	6 (0.3)	3 (0.3)	3 (0.3)	0.410
Prior OAC	1,472 (73.6)	721 (74.0)	751 (73.2)	0.248
Prior VKA	54 (2.7)	23 (2.4)	31 (3.0)	0.359
Prior NOAC	1,418 (70.9)	698 (71.7)	720 (70.2)	0.138
Apixaban	851 (42.6)	447 (45.9)	404 (39.4)	0.001
Dabigatran	99 (5.0)	51 (5.2)	48 (4.7)	0.564
Edoxaban	319 (16.0)	134 (13.8)	185 (18.0)	0.007
Rivaroxaban	149 (7.5)	66 (6.8)	83 (8.1)	0.256
Atrial fibrillation type				
Not determined	83 (4.2)	32 (3.3)	51 (5.0)	0.059
Paroxysmal	1,044 (52.2)	516 (53.0)	528 (51.5)	0.498
Non paroxysmal	873 (43.7)	426 (43.6)	447 (43.6)	0.939
Persistent	705 (35.3)	334 (34.3)	371 (36.2)	0.382
Long–standing persistent	92 (4.6)	48 (4.9)	44 (4.3)	0.495
Permanent	72 (3.6)	43 (4.4)	29 (2.8)	0.057
EHRA classification				
I	478 (23.9)	225 (23.1)	253 (24.7)	0.414
IIa	790 (39.5)	387 (49.0)	403 (39.3)	0.835
IIb	244 (12.2)	104 (10.7)	140 (13.6)	0.043
III	57 (2.9)	20 (2.1)	37 (3.6)	0.037
IV	2 (0.1)	1 (0.1)	1 (0.1)	0.971
Unknown	429 (21.4)	237 (24.3)	192 (18.7)	0.002
Lab				
Haemoglobin (g/dl)***	13.0 ± 1.8	13.0 ± 1.9	12.5 ± 1.8	<0.001
Anaemia (men ≤13 g/dl, female ≤12 g/dl)	537 (26.9)	229 (23.5)	308 (30.0)	<0.001
Platelet (×10^3^/μl)	207.8 ± 69.2	209.1 ± 74.8	208.0 ± 69.0	0.174
PT INR****	1.2 ± 0.5	1.2 ± 0.3	1.2 ± 0.7	0.938
Creatinine (mg/dL)	1.0 ± 0.7	0.9 ± 0.8	1.1 ± 0.9	<0.001
CrCl (ml/min)	56.2 ± 17.4	59.5 ± 17.2	53.4 ± 19.0	<0.001
CrCl <50 (ml/min)	733 (36.7)	276 (28.3)	457 (44.5)	<0.001
eGFR (MDRD) (ml/min/1.73m^2^)	72.4 ± 23.0	77.3 ± 23.0	70.4 ± 26.5	<0.001
eGFR (CKD–EPI) (mL/min/1.73m^2^)	71.0 ± 19.9	75.3 ± 18.2	67.8 ± 21.2	<0.001
Stage 1 (eGFR ≥90)	276 (13.8)	185 (19.0)	91 (8.9)	<0.001
Stage 2 (60 ≤eGFR <90)	1,123 (56.2)	578 (59.3)	545 (53.1)	0.025
Stage 3a (45 ≤eGFR <60)	344 (17.2)	137 (14.1)	207 (20.2)	<0.001
Stage 3b (30 ≤eGFR <45)	156 (7.8)	55 (5.6)	101 (9.8)	<0.001
Stage 4 (15 ≤eGFR <30)	42 (2.1)	6 (0.6)	36 (3.5)	<0.001
Stage 5 (eGFR <15)	15 (0.8)	3 (0.3)	12 (1.2)	0.023

Categorical variables were presented as a percentage and continuous variables were presented as mean and standard deviation.

CKD, chronic kidney disease; CrCl, Creatinine clearance; DAPT, dual antiplatelet therapy; eGFR, estimated glomerular filtration rate; EHRA, European Heart Rhythm Association; MDRD, modification of diet in renal disease; NOAC, Non–Vitamin K antagonist oral anticoagulants; PT INR, prothrombin time international normalized ratio; SAPT, single antiplatelet therapy; TIA, transient ischemic attack; VKA, Vitamin K antagonist.

* *N* = 1,934.

** *N* = 1,939.

*** *N* = 1,532.

**** *N* = 544.

### Baseline characteristics according to apixaban dose in subgroups with three different dose reduction criteria

**Figure 2:**
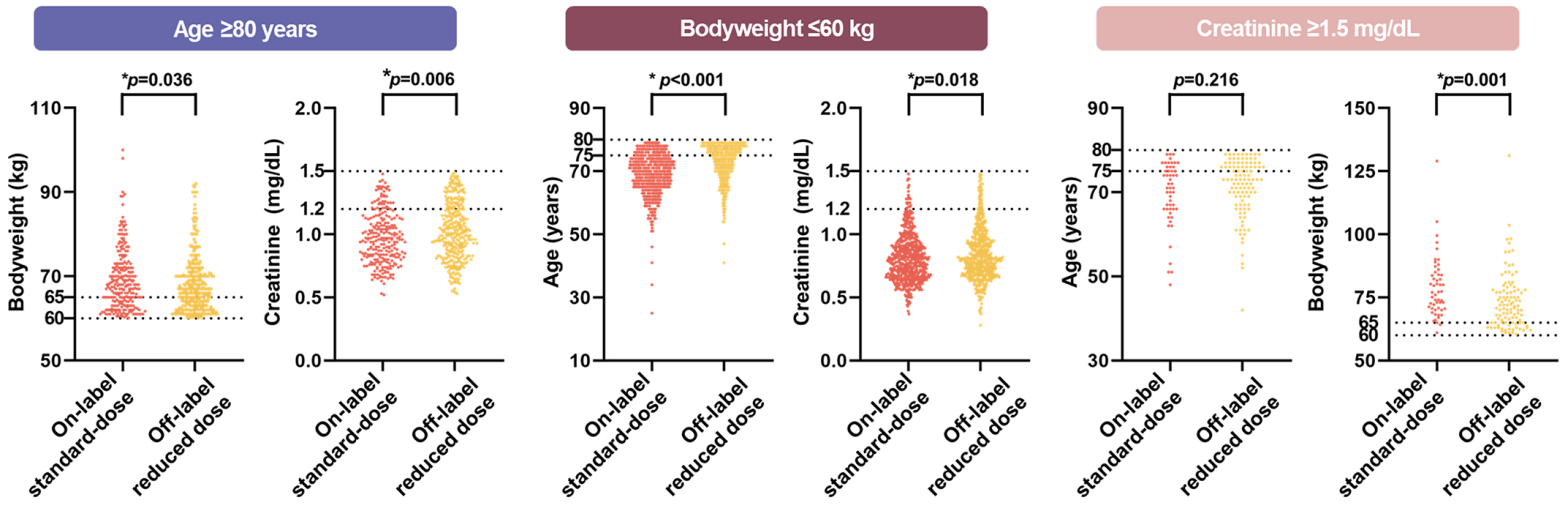
Proportion of on-label standard-dose and off-label reduced in each inclusion criteria. The doughnut charts show the proportion of on-label standard-dose and off-label reduced in each inclusion criteria (total, age ≥80 years, body weight ≤60 kg, and serum creatinine ≥1.5 mg/dL). More than half of total population were prescribed with off-label reduced dose apixaban.

The apixaban dosing pattern in each subgroup according to dose reduction criteria (age, body weight, and serum creatinine level) is presented in Figure 2. Of 593 patients in the age ≥80 years group, 41.7% (*n* = 247) were receiving on-label standard-dose apixaban and 58.3% (*n* = 346) were receiving off-label reduced apixaban. In participants with body weight ≤60 kg, 54.0% (*n* = 675) of them were receiving on-label standard-dose and 46.0% (*n* = 576) were receiving off-label reduced dose. Among 156 patients with serum creatinine ≥1.5 mg/dL, 33.3% (*n* = 52) were receiving on-label standard-dose, whereas 66.7% (*n* = 104) were receiving off-label reduced dose. Among three dose reduction criteria, patients with low body weight (≤60 kg) had the highest proportion of on-label standard-dose apixaban prescription and patients with renal impairment (serum creatinine ≥1.5 mg/dL) had the lowest proportion of on-label standard-dose apixaban prescription.

The baseline characteristics of each dose-reduction criterion according to the apixaban dose group are described in Supplementary Table S2. In all three dose reduction criteria, the off-label reduced dose group was older, had lower body weight, and had significantly lower kidney function than the on-label standard-dose group. Patients in the off-label reduced dose group were more likely to be women in the age ≥80 years or body weight ≤60 kg groups.

### Distribution of other two dose reduction components in patients who met a single criterion for dose reduction

**Figure 3:**
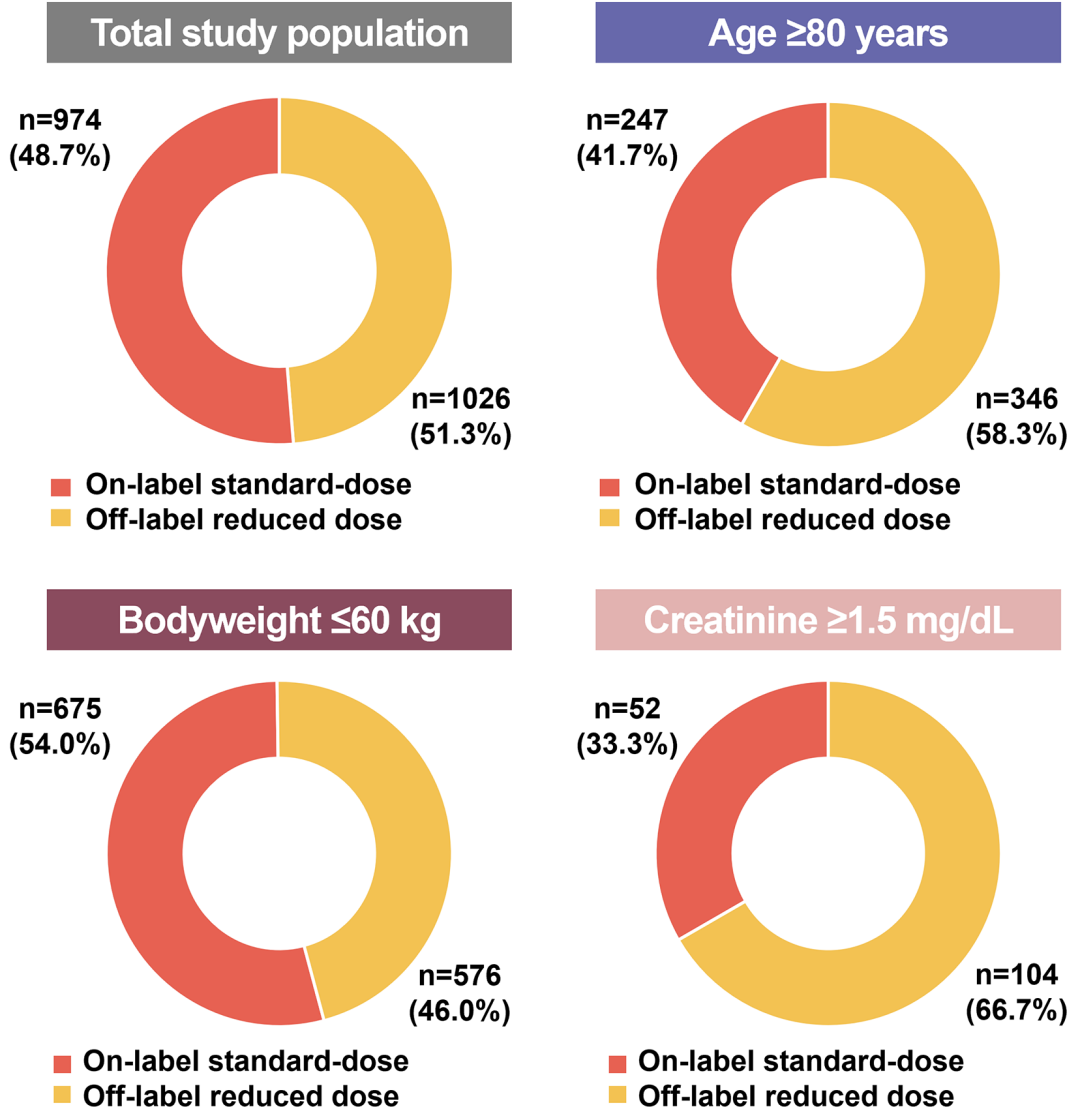
The violin plot of additional dose reduction criteria in each inclusion criteria. Among all inclusion criteria groups, the off-label reduced group was more likely to be distributed in the marginal zone.

The proportions of the “marginal zone” of two dose reduction components other than the index dose reduction component showed different distributions among the on-label standard-dose group and off-label reduced dose group (Supplementary Table S2 and Figure 3). In the age ≥80 years group, the serum creatinine levels between 1.2 and 1.5 mg/dL were more prevalent in off-label reduced dose group (*p *= 0.013). In the bodyweight ≤60 kg group, the prevalence of both age (between 75 and 80 years) and serum creatinine level (between 1.2 and 1.5 mg/dL) was higher in the off-label reduced dose group (*p *< 0.001 and *p *= 0.021, respectively). In the creatinine ≥1.5 mg/dL group, body weight between 60 and 65 kg was more common in off-label reduced dose group (*p *= 0.001). A scatter plot of the remaining inclusion criteria values for each inclusion criterion is shown in Figure 3. Among all inclusion criteria groups, the off-label reduced group was more likely to be distributed in the marginal zone. This trend was more pronounced in the areas where the two marginal zones overlapped (Supplementary Figure S1).

### Baseline characteristics of on-label, off-label marginal, and off-label non-marginal groups

The baseline characteristics comparison of on-label standard-dose, marginal off-label reduced dose, and non-marginal off-label reduced dose groups are described in Supplementary Table S3. The marginal off-label reduced dose group was the oldest (mean age 72.7 ± 8.1 years in the on-label standard-dose group, 78.4 ± 4.9 years in marginal off-label reduced dose group, and 73.8 ± 8.1 years in non-marginal off-label reduced dose, *p *< 0.001) with the highest age marginal zone (75–79 years old) proportion (16.9% in 5 mg on-label standard-dose group, 56.0% in marginal off-label reduced dose group, and 9.1% in non-marginal off-label reduced dose, *p *< 0.001) compared to other groups. They also had the highest mean CHA₂DS₂-VASc (3.2 ± 1.2 in on-label standard-dose group, 3.8 ± 1.1 in marginal off-label reduced dose group, and 3.2 ± 1.1 in non-marginal off-label reduced dose, *p *< 0.001) and HAS-BLED scores (1.6 ± 0.9 in on-label standard-dose group, 1.7 ± 0.8 in marginal off-label reduced dose group, and 1.6 ± 0.9 in non-marginal off-label reduced dose, *p *< 0.015) compared to other groups. Those in the marginal off-label reduced dose group had the lowest mean haemoglobin level (13.0 ± 1.9 g/dl in the on-label standard-dose group, 12.3 ± 1.8 g/dl in the marginal off-label reduced dose group, and 12.7 ± 1.8 g/dl in non-marginal off-label reduced dose, *p *< 0.001) with the highest anaemia proportion (23.5% in on-label standard-dose group, 32.9% in marginal off-label reduced dose group, and 27.5% in non-marginal off-label reduced dose, *p *< 0.001).

### Factors associated with off-label reduced dose apixaban prescription

Factors significantly associated with off-label reduced dose apixaban prescriptions are summarised in Table 2. In the total population, age and serum creatinine, considered as continuous variables, were significantly associated with off-label dosing. Among the patients aged ≥80 years, serum creatinine levels between 1.2–1.5 mg/dL and women, associated with a higher prevalence of off-label reduced dose (*p *= 0.004 and *p *= 0.010, respectively). In the bodyweight ≤60 kg group, age between 75 and 79 years and concomitant APT use remained positively associated with off-label reduced dose (*p *< 0.001 and *p *< 0.012, respectively). Previous history of stroke/TIA was negatively associated with off-label reduced dose (*p *= 0.001). In the serum creatinine ≥1.5 mg/dL group, bodyweight between >60 and 65 kg remained significantly associated with off-label reduced dose in multivariable logistic regression (*p *= 0.006).

**Table 2:** Factors associated with Off–label reduced dose of apixaban in each inclusion criteria.

	Univariable		Multivariable	
	OR (95% CI)	*p-*value	OR (95% CI)	*p-*value
Total				
Age	1.064 (1.051–1.077)	<0.001	** **	** **
Bodyweight	1.006 (0.997–1.015)	0.209	** **	** **
Cr	1.675 (1.334–2.103)	<0.001	** **	** **
Age ≥80 years				
Bodyweight 60–65 kg	1.251 (0.893–1.751)	0.193		
Serum creatinine 1.2–1.5 mg/dL	1.688 (1.116–2.553)	0.013	1.870 (1.225–2.854)	0.004
Female	1.468 (1.024–2.103)	0.037	1.626 (1.125–2.350)	0.01
Hypertension	1.210 (0.807–1.813)	0.357		
Previous stroke/TIA	0.638 (0.382–1.067)	0.087		
Previous bleeding	0.947 (0.543–1.652)	0.848		
Antiplatelet use	1.560 (0.808–3.010)	0.185		
Anaemia	1.382 (0.932–2.048)	0.107		
Bodyweight ≤60 kg				
Age 75–79 years	3.487 (2.730–4.455)	<0.001	3.205 (2.383**–**4.310)	<0.001
Serum creatinine 1.2–1.5 mg/dL	1.791 (1.085–2.957)	0.023	1.439 (0.785–2.638)	0.239
Female	1.131 (0.880–1.453)	0.336		
Hypertension	1.154 (0.916–1.455)	0.224		
Previous stroke/TIA	0.476 (0.318–0.713)	<0.001	0.434 (0.266–0.709)	0.001
Previous bleeding	2.188 (1.311–3.653)	0.003	1.697 (0.900–3.201)	0.102
Antiplatelet use	1.937 (1.147–3.272)	0.013	2.213 (1.194–4.103)	0.012
Anaemia	1.412 (1.074–1.858)	0.014	1.280 (0.952–1.720)	0.102
Creatinine ≥1.5 mg/dL				
Age 75–79 years	1.340 (0.665–2.699)	0.413		
Bodyweight 60–65 kg	6.316 (1.824–21.868)	0.004	5.837 (1.668–20.422)	0.006
Female	5.233 (1.165–23.495)	0.031	4.569 (0.991–21.057)	0.051
Hypertension	0.827 (0.349–1.961)	0.667		
Previous stroke/TIA	0.805 (0.326–1.987)	0.638		
Previous bleeding	1.193 (0.430–3.308)	0.735		
Antiplatelet use	1.530 (0.519–4.512)	0.441		
Anaemia	1.574 (0.751–3.298)	0.230		

CI, confidence interval; OR, odds ratio; TIA, transient ischemic attack.

## Discussion

**Figure 4:**
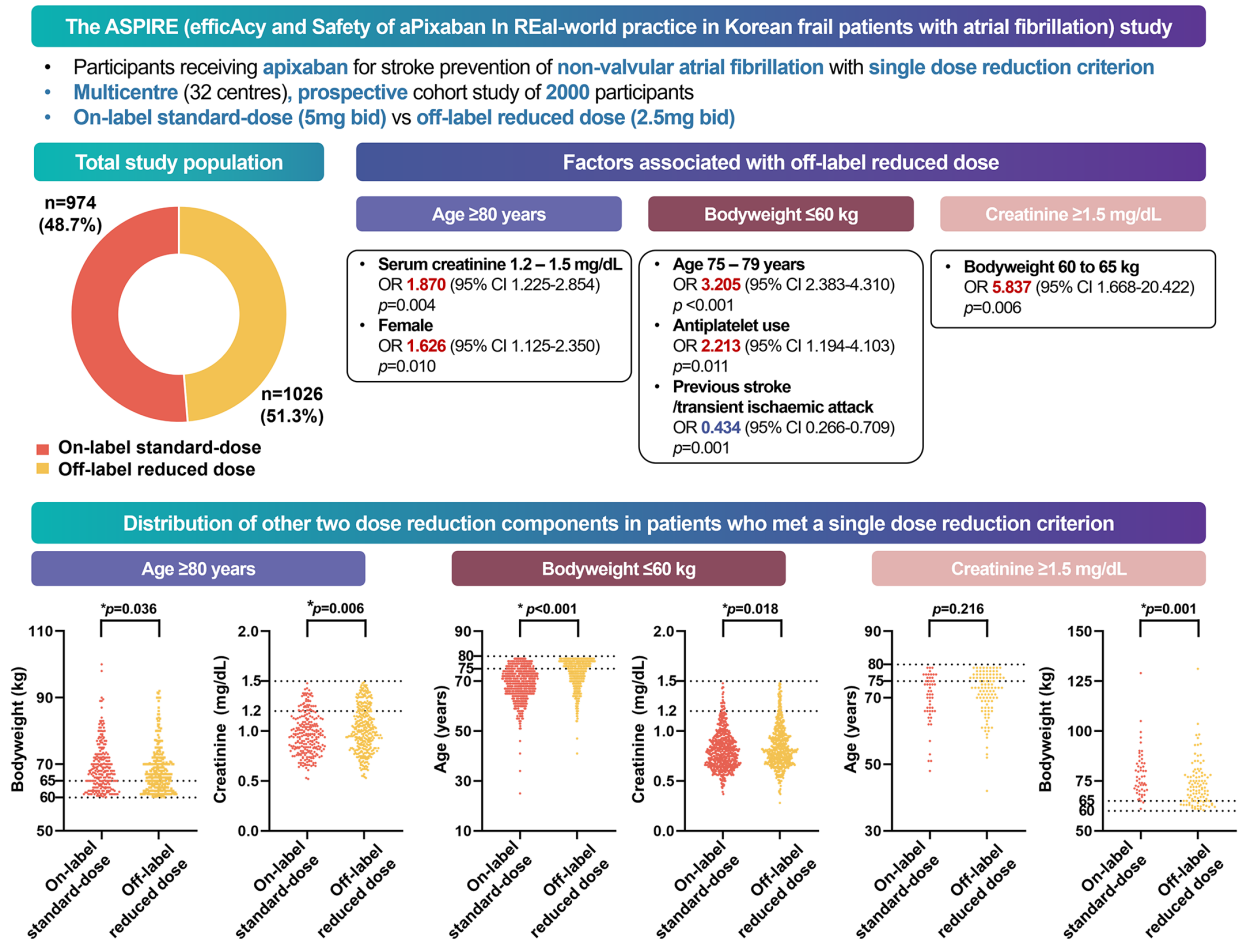
Graphical abstract of the study. In a multicenter, prospective cohort study of South Korean participants with atrial fibrillation, half of the patients who met one apixaban criterion received off-label reduced doses in real-world practice. Physicians were more likely to prescribe reduced doses to frail patients, whereas those with a history of strokes received on-label doses.

In this prospective, multicentre, non-interventional observational study of 2,000 Asian patients with a single criterion for dose reduction for apixaban, we found that (Figure 4) (1) low body weight was the most common cause of off-label dose reduction, followed by old age and renal dysfunction; (2) almost half of the patients received the off-label reduced dose apixaban; (3) the off-label reduced dose group had more hypertension, heart failure, bleeding history, CKD, anaemia, and concomitant APT; and (4) marginal values of other criteria also influenced the off-label prescription.

Currently, DOACs are considered the primary treatment option among participants with AF for stroke prevention (1–3). The optimal dose for each patient is an area of interest. Previous observational studies have shown that off-label reduced dose of DOACs is associated with an increased stroke risk (8, 11, 12). Although previous studies used different types of DOACs and study populations, dose reduction of apixaban was associated with increased stroke in both Western and Asian countries (8, 11). Thus, prescribing an on-label dose of apixaban is strongly recommended unless patients fulfil more than two dose reduction criteria (5, 13).

Despite the current dosing guidelines, low-dose apixaban is more likely to be prescribed to Asian populations (9, 14, 23). Among Korean patients with AF prescribed DOAC, apixaban 2.5 mg twice daily was prescribed to more patients than those prescribed 5 mg apixaban twice daily (54.1 vs. 45.9%) (14). In the COmparison study of Drugs for symptom control and complication prEvention of Atrial Fibrillation (CODE-AF) registry, a prospective multicentre registry of Korean patients with AF, the proportion of off-label reduced dose was higher in patients taking apixaban than in those taking other DOACs (9). A nationwide retrospective cohort study in Taiwan also showed a high prevalence of low-dose DOAC use (24). However, few studies have reported clinical outcomes according to the number of dose reduction criteria for apixaban. We previously reported the clinical outcomes of off-label reduced dose apixaban using the Korean nationwide claims database (12). In a previous study, participants taking off-label reduced dose apixaban, despite not fulfilling the dose reduction criteria, exhibited a twice as high risk of ischaemic stroke compared to those receiving the on-label standard dose of apixaban.” (12). However, among patients who met the single criterion for dose reduction, there was no significant difference in the risk of ischaemic stroke between off-label reduced dose and on-label standard-dose apixaban (12).

Although a previous retrospective cohort study based on the Korean nationwide claims database suggested that the clinical impact of off-label reduced dose apixaban on the risk of ischaemic stroke could be negligible in patients with a single criterion for dose reduction, we aimed to evaluate the difference between the on-label standard-dose and off-label reduced dose apixaban among participants who met single dose reduction criteria in a prospectively collected cohort, the ASPIRE study. In the current study, even though the patients only met a single criterion for dose reduction, over 50% of the study population received off-label reduced dose apixaban. One concern regarding off-label reduced dose of apixaban is whether it increases the risk of stroke.

Prior landmark clinical trials have mostly included Caucasian participants with different anthropometric measurements from the Asian population (e.g., heavier mean body weight) (13, 25–28). Thus, concerns remain regarding whether off-label reduced dose in participants who fulfil only a single criterion for dose reduction would result in benefits or harm. A *post hoc* study using the ENGAGE AF-TIMI 48 trial data showed that a lower-dose edoxaban regimen (LDER) was associated with significantly better net clinical outcomes than the higher-dose edoxaban regimen (HDER) due to less frequent major bleeding events in the Western population (29). Although the previously published ENGAGE AF-TIMI 48 trial showed a 41% increased risk of ischaemic stroke in patients with LDER (25), a recent analysis supports that reduced dose may be a feasible alternative option for patients at high bleeding risk. Similarly, the Edoxaban Low-Dose for Elder Care Atrial Fibrillation Patients (ELDERCARE-AF) trial showed that low-dose edoxaban 15 mg in older adults with bleeding risk factors was beneficial for stroke prevention compared to placebo, with no significant increase in major bleeding in the Japanese population (30).

Pharmacokinetic and pharmacodynamic studies of rivaroxaban in Asian populations indicate higher apparent clearance in Caucasians than in Asians, suggesting that a lower dose might offer comparable thromboembolic event protection in Asians as the standard dose does in Caucasians (31). In a small study that monitored the plasma concentrations of rivaroxaban and apixaban in Asian participants, off-label reduced dose showed an appropriate plasma concentration with no bleeding or thromboembolic events (32). These studies underscore the potential need for dose adjustments based on ethnicity and patient-specific factors.

In previous studies, factors such as age, renal dysfunction, past medical history (prior bleeding, hypertension, and congestive heart failure), anaemia, and concomitant APT were found to affect the physicians' prescription of a reduced dose of DOACs (33–35). In our study, the off-label reduced dose group had more comorbidities, such as hypertension, heart failure, and a history of bleeding, suggesting that physicians might be prescribing reduced doses to mitigate bleeding risks. This practice reflects the delicate balance clinicians must maintain between preventing thromboembolic events and minimizing bleeding risks in frail populations. The three dose reduction criteria for apixaban were not completely exclusive in this study. Although the participants only fully satisfied the single criterion for dose reduction and no other criteria, they were likely to have marginal zone values for the other two dose reduction criteria. Again, such accompanying “marginal zone” factors would have influenced clinicians in prescribing off-label reduced dose apixaban rather than using the on-label standard-dose apixaban. We also assessed additional factors other than the dose reduction criteria that affected the physician in prescribing off-label reduced dose apixaban. In participants aged ≥80 years, female sex significantly affected the physician in choosing a lower dose. In those with a body weight of ≤60 kg, concomitant APT was significantly associated with reduced dose. These factors overlapped with previously reported factors associated with off-label reduced dose (33–35). Therefore, physicians are likely to adopt a lower dose of apixaban for patients with a marginal value of additional dose reduction criteria or other factors that represent frailty.

The apixaban prescription data shown in this study depict the biggest concern that Korean clinicians face in real-life clinical settings, that is, low body weight. This concern arises from the finding that underweight Korean patients with AF receiving OAC have a higher risk of experiencing various clinical events, such as stroke, intracranial haemorrhage, gastrointestinal bleeding, major bleeding, and all-cause death than those with normal body weight (36). Owing to concerns about low body weight, the safety and effectiveness of DOACs have been addressed in low (body weight of ≤60 kg) and very low (body weight <50 kg) body weight patients, resulting in DOACs being a safer and more effective option than warfarin (28). Furthermore, the ENGAGE AF-TIMI 48 trial showed that patients with body weight ≤60 kg may benefit from a lower dose of edoxaban in relation to bleeding events (37). However, the outcome of apixaban off-label reduced dose in patients with only one dose-reduction criterion has not been addressed. Further analysis of the clinical outcomes of these apixaban off-label reduced dose groups will provide an overall insight into the feasibility of an off-label reduced dose of apixaban in the Korean population.

Although this study was not an RCT, it included data from 2,000 Asians with a single frail component and an off-label reduced prescription. As a previous pivotal RCT showed prescription data of reduced dose apixaban among less than 500 participants, our study has strength in a larger real-world study population (13).

### Study limitations

There were some limitations to this study. First, this was not an RCT but a non-interventional observational design. Selection of the apixaban dose was solely at the physician's discretion. However, such an observational design would best assess the factors that affect physicians' decisions on apixaban dose in real-world clinical practice beyond current clinical guidelines. Second, generalisability should be considered cautiously, as the study population included only East Asians, specifically Koreans. Third, this study only described the baseline characteristics of different apixaban dose groups and not the subsequent clinical outcomes related to dosage differences, which will be reported in a subsequent analysis.

## Conclusions

In practical clinical settings within the Korean population, nearly 50% of patients who met a single criterion for dose reduction of apixaban received an off-label reduced dose of the drug. Korean physicians tended to prescribe this off-label reduced dose to patients exhibiting more signs of frailty, while those with a history of stroke were more likely to receive the on-label standard dose. Further analysis of the clinical outcomes in these apixaban off-label reduced dose groups will provide deeper insights into the feasibility and safety of such practices, especially in the Korean population.

## Data Availability

The raw data supporting the conclusions of this article will be made available by the authors, without undue reservation.
