# Supplementary material for: Clinical characteristics of apixaban prescription in AF patients with single dose-reduction criterion: the ASPIRE (efficAcy and safety of aPixaban in rEal-world practice in Korean frail patients with atrial fibrillation) study
Source: Front Cardiovasc Med. 2024 Jun 10;11:1367623. doi: 10.3389/fcvm.2024.1367623 (PMC11194408; doi:10.3389/fcvm.2024.1367623)

**Supplementary Materials**

**Supplemental Tables**

**Table S1.** ASPIRE sites, investigators, and affiliations.

**Table S2.** Baseline characteristics according to each inclusion criteria.

**Table S3.** Baseline characteristics of the apixaban on-label standard-dose, marginal off-label reduced dose group, and non-marginal off-label reduced dose group.

**Supplemental Figures**

**Figure S1.** Distribution of subjects in each criterion according to remaining two criteria.

**Table S1. ASPIRE sites, investigators, and affiliations**

| **Site** | **Investigator names and academic degree** | **Affiliations** |
| --- | --- | --- |
| Kyung Hee University Hospital at Gangdong | Eun-Sun Jin, MD, PhD | Department of Cardiology, Kyung Hee University College of Medicine, Kyung Hee University Hospital at Gangdong, Seoul |
| Kangbuk Samsung Hospital | Sung Ho Lee, MD, PhD | Division of Cardiology, Department of Internal Medicine, Kangbuk Samsung Hospital, Sungkyunkwan University School of Medicine, Seoul |
| Kyung-Hee University Hospital | Jin-Bai Kim, MD, PhD | Division of Cardiology, Department of Internal Medicine, Kyung-Hee University Hospital, Kyung-Hee University, Seoul |
| Keimyung University Dongsan Hospital | Seongwook Han, MD, PhD | Division of Cardiology, Department of Internal Medicine, Keimyung University Dongsan Hospital, Daegu |
|  | Hyoung-Seob Park, MD | Division of Cardiology, Department of Internal Medicine, Keimyung University Dongsan Hospital, Daegu |
|  | Jongmin Hwang, MD, PhD | Division of Cardiology, Department of Internal Medicine, Keimyung University Dongsan Hospital, Daegu |
| Korea University Guro Hospital | Seung-Young Roh, MD, PhD | Department of Internal Medicine, Korea University College of Medicine, Seoul |
| Korea University Anam Hospital | Jong-Il Choi, MD, PhD, MHS, MS | Department of Internal Medicine, Division of Cardiology, Korea University College of Medicine and Korea University Anam Hospital, Seoul |
| Kosin University Gospel Hospital | Jung Ho Heo, MD, PhD | Department of Internal Medicine, Kosin University Gospel Hospital, Busan |
| National Health Insurance Service Ilsan Hospital | Jeon Dong Woon, MD, PhD | Department of Internal Medicine, National Health Insurance Service Ilsan Hospital, Gyeonggi |
| Dongguk University Ilsan Hospital | Ungjeong Do, MD | Division of Cardiology, Department of Internal Medicine, Dongguk University Ilsan Hospital, Goyang, Korea |
| Dong-A University Hospital | Jong-Sung Park, MD, PhD | Department of Cardiology, Dong-A University Hospital, Busan |
|  | Kyung Hee Lim, MD, PhD | Department of Cardiology, Dong-A University Hospital, Busan |
| Seoul National University Hospital | Eue-Keun Choi, MD, PhD | Department of Internal Medicine, Seoul National University Hospital, Seoul |
|  | So‑Ryoung Lee, MD, PhD | Department of Internal Medicine, Seoul National University Hospital, Seoul |
|  | Seil Oh, MD, PhD | Department of Internal Medicine, Seoul National University Hospital, Seoul |
| Seoul National University Bundang Hospital | Il-Young OH, MD, Ph.D | Department of Internal Medicine, Seoul National University Bundang Hospital, Gyeonggi |
|  | Cho, Youngjin, MD, PhD | Division of Cardiology, Seoul National University Bundang Hospital, Gyeonggi |
|  | Lee, Jihyun, MD, PhD | Division of Cardiology, Seoul National University Bundang Hospital, Gyeonggi |
| Samsung Medical Center | Young Keun On, MD, PhD | Department of Cardiology, Heart Vascular Stroke Institute, Samsung Medical Center, Sungkyunkwan University School of Medicine, Seoul |
| Samsung Changwon Hospital | Hye Bin Gwag, MD, PhD | Department of Internal Medicine, Samsung Changwon Hospital, Sungkyunkwan University School of Medicine, Changwon |
| SMG-SNU Boramae Medical Center | Woo-Hyun Lim, MD | Department of Internal Medicine, SMG-SNU Boramae Medical Center, Seoul |
| Asan Medical Center | Kee-Joon Choi, MD, PhD | Department of Internal Medicine, Asan Medical Center, Ulsan University, Seoul |
| Soonchunhyang University Cheonan Hospital | Seung-Jin Lee, MD, PhD | Department of Internal Medicine, Soonchunhyang University Cheonan Hospital, Cheonan |
| Severance Cardiovascular Hospital | Hee Tae Yu, MD, PhD | Department of Internal Medicine, Severance Cardiovascular Hospital, Yonsei University College of Medicine, Seoul |
|  | Tae-Hoon Kim, MD | Department of Internal Medicine, Severance Cardiovascular Hospital, Yonsei University College of Medicine, Seoul |
|  | Daehoon Kim, MD | Department of Internal Medicine, Severance Cardiovascular Hospital, Yonsei University College of Medicine, Seoul |
| Ajou University Hospital | Kwang-No Lee, MD, PhD | Department of Cardiology, Ajou University School of Medicine, Suwon |
| Andong General Hospital | Dae-Woo Hyun, MD, PhD | Department of Cardiovascular Center, Andong General Hospiral, Andong |
| Yeungnam University Hospital | Dong-Gu Shin, MD, PhD | Department of Cardiovascular Division, Yeungnam University Hospital, Daegu |
| Wonkwang University Hospital | Nam-Ho Kim, MD, PhD | Department of Internal Medicine, Wonkwang University Hospital, Iksan |
|  | Kyeong Ho Yun, MD, PhD | Department of Internal Medicine, Wonkwang University Hospital, Iksan |
| Inje University Haeundae Paik Hospital | Sang-Hoon Seol, MD, PhD | Department of Internal Medicine, Inje University Haeundae Paik Hospital, Busan |
| Inje University Pusan Paik Hospital | Dae-Kyeong Kim, MD, PhD | Department of Internal Medicine, Inje University Pusan Paik Hospital, Busan |
| Inje University Ilsan Paik Hospital | June Namgung, MD, PhD | Department of Internal Medicine, Inje University Ilsan Paik Hospital, Goyang-si |
| Chonnam National University Hospital | Hyung Wook Park, MD, PhD | Department of Cardiovascular Medicine, Chonnam National University Hospital, Gwangju |
| Chonnam National University Hospital | Ki Hong Lee, MD, PhD, FESC, FKHRS, | Department of Cardiovascular medicine, Chonnam National University Hospital, Gwangju |
| Jeonbuk National University Hospital | Kyoung-Suk Rhee, MD, PhD | Department of Internal Medicine, Jeonbuk National University Hospital, Jeonbuk |
| Jeju National University hospital | Joon Hyouk Choi, MD, PhD | Department of Cardiology, Jeju national university hospital, Jeju |
| Chung-Ang University Hospital | Seung Yong Shin, MD, PhD | Cardiovascular & Arrhythmia center, Chung-Ang University Hospital, Seoul |
| Hanyang University Guri Hospital | Hwan-Cheol Park MD, PhD | Division of Cardiology, Department of Internal Medicine, Hanyang University College of Medicine, Gyeonggi |
| Hanyang University Medical Center | Jin-Kyu Park, MD, PhD | Department of Internal Medicine, Hanyang University Medical Center, Seoul |

**Table S2. Baseline characteristics according to each inclusion criteria.**

**(A) Age ≥80 years**

|  | **Total**  **(n=593)** | **Apixaban dose** | | ***p*-value** |
| --- | --- | --- | --- | --- |
|  |  | **On-label  standard-dose**  **(n=247)** | **Off-label  reduced dose**  **(n=346)** |  |
| **Age, years** | 82.6 ± 2.6 | 82.2 ± 2.5 | 83.1 ± 3.4 | 0.009 |
| **Sex (female)** | 184 (31.0) | 65 (26.3) | 119 (34.4) | 0.036 |
| **Bodyweight (kg)** | 68.5 ± 6.8 | 69.1 ± 7.1 | 68.0 ± 6.6 | 0.049 |
| **60–65** | 231 (39.0) | 89 (36.0) | 142 (41.0) | 0.207 |
| **CHA₂DS₂-VASc score** | 3.8 ± 1.0 | 3.9 ± 0.9 | 4.0 ± 1.1 | 0.023 |
| **≥3** | 547 (92.2) | 226 (91.5) | 321 (92.8) | 0.567 |
| **HAS-BLED score ^*^** | 1.8 ± 0.8 | 2.1 ± 0.9 | 2.0 ± 0.9 | 0.510 |
| **≥3** | 86 (14.5) | 44 (17.8) | 42 (12.1) | 0.093 |
| **Comorbidities** |  |  |  |  |
| **Hypertension** | 474 (79.9) | 193 (78.1) | 281 (81.2) | 0.356 |
| **Diabetes Mellitus** | 201 (33.9) | 79 (32.0) | 122 (35.3) | 0.406 |
| **Heart failure** | 148 (25.0) | 52 (21.1) | 96 (27.7) | 0.063 |
| **Prior stroke/TIA** | 66 (11.1) | 34 (13.8) | 32 (9.2) | 0.085 |
| **Prior bleeding** | 56 (9.4) | 24 (9.7) | 32 (9.2) | 0.848 |
| **CKD** | 57 (9.6) | 12 (4.9) | 45 (13.0) | 0.001 |
| **Not on dialysis** | 56 (9.4) | 11 (4.5) | 45 (13.0) | 0.211 |
| **On dialysis** | 1 (0.2) | 0 (0.0) | 1 (0.3) | 0.211 |
| **Transplantation** | 0 (0.0) | 0 (0.0) | 0 (0.0) |  |
| **Liver disease** | 21 (3.5) | 9 (3.6) | 12 (3.5) | 0.909 |
| **Malignancy** | 88 (14.8) | 37 (15.0) | 51 (14.7) | 0.935 |
| **Antiplatelet use^**^** | 44 (7.4) | 14 (5.7) | 27 (7.8) | 0.182 |
| **SAPT** | 40 (6.7) | 14 (5.7) | 26 (7.5) | 0.290 |
| **DAPT** | 1 (0.2) | 0 (0.0) | 1 (0.3) | 1.000 |
| **Prior OAC** | 443 (74.7) | 184 (74.5) | 259 (74.9) | 0.650 |
| **Prior VKA** | 16 (2.7) | 6 (2.4) | 10 (2.9) | 0.726 |
| **Prior NOAC** | 427 (72.0) | 178 (72.1) | 249 (72.0) | 0.934 |
| **Apixaban** | 270 (45.5) | 114 (46.2) | 156 (45.1) | 0.802 |
| **Dabigatran** | 40 (6.7) | 23 (9.3) | 17 (4.9) | 0.034 |
| **Edoxaban** | 75 (12.6) | 25 (10.1) | 50 (14.5) | 0.107 |
| **Rivaroxaban** | 42 (7.1) | 16 (6.5) | 26 (7.5) | 0.615 |
| **Atrial fibrillation type** |  |  |  |  |
| **Not determined** | 31 (5.2) | 14 (5.7) | 17 (4.9) | 0.684 |
| **Paroxysmal** | 272 (45.9) | 107 (43.3) | 165 (47.7) | 0.293 |
| **Non paroxysmal** | 290 (48.9) | 126 (51.0) | 164 (47.4) | 0.386 |
| **Persistent** | 225 (37.9) | 93 (37.7) | 132 (38.2) | 0.145 |
| **Long-standing persistent** | 35 (5.9) | 15 (6.1) | 20 (5.8) | 0.925 |
| **Permanent** | 29 (4.9) | 18 (7.3) | 11 (3.2) | 0.034 |
| **EHRA classification** |  |  |  |  |
| **I** | 179 (30.2) | 54 (26.3) | 114 (32.9) | 0.083 |
| **IIa** | 185 (31.2) | 92 (32.7) | 93 (26.9) | 0.007 |
| **IIb** | 66 (11.1) | 25 (10.1) | 41 (11.8) | 0.509 |
| **III** | 19 (3.2) | 5 (2.0) | 14 (4.0) | 0.168 |
| **IV** | 1 (0.2) | 0 (0.0) | 1 (0.3) | 1.000 |
| **Unknown** | 143 (24.1) | 60 (24.3) | 83 (24.0) | 0.932 |
| **Lab** |  |  |  |  |
| **Haemoglobin (g/dL) ^***^** | 13.2 ± 1.9 | 13.3 ± 2.0 | 12.3 ± 2.0 | 0.002 |
| **Anemia (men ≤13 g/dL, female ≤12 g/dL)** | 180 (30.4) | 59 (23.9) | 101 (29.2) | 0.107 |
| **Platelet (x10^3^/μl)** | 196.3 ± 62.4 | 185.0 ± 49.8 | 201.8 ± 65.6 | 0.929 |
| **PT INR ^****^** | 1.2 ± 0.4 | 1.2 ± 0.3 | 1.2 ± 0.4 | 0.681 |
| **Creatinine (mg/dL)** | 1.0 ± 0.2 | 0.9 ± 0.2 | 1.0 ± 0.2 | 0.007 |
| **1.2–1.5** | 128 (21.6) | 41 (16.6) | 87 (25.1) | 0.013 |
| **CrCl (ml/min)** | 54.6 ± 13.4 | 58.6 ± 13.6 | 52.7 ± 13.5 | <0.001 |
| **CrCl <50** | 233 (39.3) | 77 (31.2) | 156 (45.1) | <0.001 |
| **eGFR (MDRD) (mL/min/1.73m^2^)** | 64.5 ± 17.2 | 73.8 ± 16.3 | 68.0 ± 18.4 | <0.001 |
| **eGFR (CKD-EPI) (mL/min/1.73m^2^)** | 69.5 ± 17.2 | 70.3 ± 13.7 | 64.2 ± 14.9 | <0.001 |
| **Stage 1 (eGFR ≥90)** | 8 (1.3) | 3 (1.2) | 5 (1.4) | 0.791 |
| **Stage 2 (60 ≤eGFR <90)** | 356 (60.2) | 171 (69.2) | 185 (53.5) | <0.001 |
| **Stage 3a (45 ≤eGFR <60)** | 180 (30.4) | 63 (25.5) | 117 (33.8) | 0.02 |
| **Stage 3b (30 ≤eGFR <45)** | 38 (6.4) | 8 (3.2) | 30 (8.7) | 0.007 |
| **Stage 4 (15 ≤eGFR <30)** | 0 (0.0) | 0 (0.0) | 0 (0.0) |  |
| **Stage 5 (eGFR <15)** | 0 (0.0) | 0 (0.0) | 0 (0.0) |  |

* N= 576

** N= 571

*** N= 460

**** N=151

Categorical variables were presented as a percentage and continuous variables were presented as mean and standard deviation.
Abbreviation: CKD, chronic kidney disease; CrCl, Creatinine clearance; DAPT, dual antiplatelet therapy; eGFR, estimated glomerular filtration rate; EHRA, European Heart Rhythm Association; MDRD, modification of diet in renal disease; NOAC, Non-Vitamin K antagonist oral anticoagulants; PT INR, prothrombin time international normalized ratio; SAPT, single antiplatelet therapy; TIA, transient ischemic attack; VKA, Vitamin K antagonist.

**(B) Bodyweight ≤60 kg**

|  | **Total**  **(n=1251)** | **Apixaban dose** | | ***p*-value** |
| --- | --- | --- | --- | --- |
|  |  | **On-label standard-dose**  **(n=675)** | **Off-label reduced dose**  **(n=576)** |  |
| **Age, years** | 70.8 ± 6.6 | 69.5 ± 6.1 | 72.1 ± 6.3 | <0.001 |
| **<65** | 199 (15.9) | 143 (21.2) | 57 (9.9) | <0.001 |
| **65–74** | 619 (49.5) | 385 (57.0) | 234 (40.6) | <0.001 |
| **75–79** | 433 (34.6) | 148 (21.9) | 285 (49.5) | <0.001 |
| **Sex (female)** | 911 (72.8) | 484 (71.7) | 427 (74.1) | 0.336 |
| **Bodyweight (kg)** | 54.2 ± 4.8 | 54.7 ± 4.6 | 53.4 ± 5.1 | <0.001 |
| **<50** | 253 (20.2) | 107 (15.9) | 146 (25.3) | <0.001 |
| **51–60** | 998 (79.8) | 568 (84.1) | 430 (74.7) | <0.001 |
| **CHA₂DS₂-VASc score** | 3.1 ± 1.2 | 2.9 ±1.3 | 3.2 ± 1.2 | <0.001 |
| **≥3** | 832 (66.5) | 402 (59.6) | 430 (74.7) | <0.001 |
| **HAS-BLED score^*^** | 1.5 ± 0.9 | 1.7 ± 1.0 | 1.8 ± 1.0 | 0.022 |
| **≥3** | 138 (11.0) | 67 (9.9) | 71 (12.3) | 0.159 |
| **Comorbidities** |  |  |  |  |
| **Hypertension** | 792 (63.3) | 417 (61.8) | 375 (65.1) | 0.224 |
| **Diabetes Mellitus** | 317 (25.3) | 179 (26.5) | 138 (24.0) | 0.299 |
| **Heart failure** | 280 (22.4) | 136 (20.1) | 144 (25.0) | 0.040 |
| **Prior stroke/TIA** | 122 (9.8) | 85 (12.6) | 37 (6.4) | <0.001 |
| **Prior bleeding** | 67 (5.4) | 24 (3.6) | 43 (7.5) | 0.002 |
| **CKD** | 52 (4.2) | 19 (2.8) | 33 (5.7) | 0.010 |
| **Not on dialysis** | 35 (2.8) | 14 (2.1) | 21 (3.6) | 0.457 |
| **On dialysis** | 4 (0.3) | 3 (0.4) | 1 (0.2) | 0.132 |
| **Previous kidney transplantation** | 13 (1.0) | 2 (0.3) | 11 (1.9) | 0.099 |
| **Liver disease** | 50 (4.0) | 25 (3.7) | 25 (4.3) | 0.567 |
| **Malignancy** | 153 (12.2) | 77 (11.4) | 76 (13.2) | 0.336 |
| **Antiplatelet use^**^** | 62 (5.0) | 24 (3.6) | 38 (6.6) | 0.012 |
| **SAPT** | 51 (4.2) | 19 (2.8) | 32 (5.6) | 0.736 |
| **DAPT** | 3 (0.2) | 2 (0.3) | 1 (0.2) | 0.554 |
| **Prior OAC** | 912 (72.9) | 494 (73.2) | 418 (72.6) | 0.036 |
| **Prior VKA** | 31 (2.5) | 14 (2.1) | 17 (3.0) | 0.240 |
| **Prior NOAC** | 881 (70.4) | 480 (71.1) | 401 (69.6) | 0.030 |
| **Apixaban** | 512 (40.9) | 309 (45.8) | 203 (35.2) | <0.001 |
| **Dabigatran** | 55 (4.4) | 26 (3.9) | 29 (5.0) | 0.335 |
| **Edoxaban** | 227 (18.1) | 102 (15.1) | 125 (21.7) | 0.003 |
| **Rivaroxaban** | 87 (7.0) | 43 (6.4) | 44 (7.6) | 0.417 |
| **Atrial fibrillation type** |  |  |  |  |
| **Not determined** | 45 (3.6) | 14 (2.1) | 31 (5.4) | 0.002 |
| **Paroxysmal** | 706 (56.4) | 388 (57.5) | 318 (55.2) | 0.419 |
| **Non paroxysmal** | 500 (40.0) | 273 (40.4) | 227 (39.4) | 0.710 |
| **Persistent** | 413 (33.0) | 219 (32.4) | 194 (33.7) | 0.145 |
| **Long-standing persistent** | 48 (3.8) | 29 (4.3) | 19 (3.3) | 0.387 |
| **Permanent** | 38 (3.0) | 24 (3.6) | 14 (2.4) | 0.265 |
| **EHRA classification** |  |  |  |  |
| **I** | 266 (21.3) | 150 (22.2) | 116 (20.1) | 0.369 |
| **IIa** | 548 (43.8) | 277 (41.0) | 271 (47.0) | 0.033 |
| **IIb** | 155 (12.4) | 71 (10.5) | 84 (14.6) | 0.030 |
| **III** | 34 (2.7) | 14 (2.1) | 20 (3.5) | 0.130 |
| **IV** | 1 (0.1) | 1 (0.1) | 0 (0.0) | 1.000 |
| **Unknown** | 247 (19.7) | 162 (24.0) | 85 (14.8) | <0.001 |
| **Lab** |  |  |  |  |
| **Haemoglobin (g/dL) ^***^** | 12.9 ± 1.6 | 12.9 ± 1.8 | 12.7 ± 1.7 | <0.001 |
| **Anemia (men ≤13 g/dL, female ≤12 g/dL)** | 306 (24.5) | 151 (22.4) | 155 (26.9) | 0.013 |
| **Platelet (x10^3^/μl)** | 214.5 ± 70.8 | 217.9 ± 80.9 | 211.7 ± 71.4 | 0.541 |
| **PT INR ^****^** | 1.2 ± 0.6 | 1.2 ±0.3 | 1.2 ± 0.8 | 0.676 |
| **Creatinine (mg/dL)** | 0.8 ± 0.2 | 0.8 ± 0.2 | 0.8 ± 0.2 | 0.014 |
| **1.2–1.5** | 67 (5.4) | 27 (4.0) | 40 (6.9) | 0.021 |
| **CrCl (ml/min)** | 59.5 ± 17.7 | 60.9 ± 17.7 | 57.4 ±19.8 | <0.001 |
| **CrCl <50 (ml/min)** | 361 (28.9) | 157 (23.3) | 204 (35.4) | <0.001 |
| **eGFR (MDRD) (mL/min/1.73m^2^)** | 78.9 ± 21.1 | 80.4 ± 23.1 | 78.6 ± 25.1 | <0.001 |
| **eGFR (CKD-EPI) (mL/min/1.73m^2^)** | 78.3 ± 16.5 | 78.9 ± 16.8 | 76.2 ± 16.8 | <0.001 |
| **Stage 1 (eGFR ≥90)** | 268 (21.4) | 182 (27.0) | 86 (14.9) | <0.001 |
| **Stage 2 (60 ≤eGFR <90)** | 767 (61.3) | 407 (60.3) | 360 (62.5) | 0.131 |
| **Stage 3a (45 ≤eGFR <60)** | 155 (12.4) | 69 (10.2) | 86 (14.9) | 0.006 |
| **Stage 3b (30 ≤eGFR <45)** | 29 (2.3) | 9 (1.3) | 20 (3.5) | 0.01 |
| **Stage 4 (15 ≤eGFR <30)** | 0 (0.0) | 0 (0.0) | 0 (0.0) |  |
| **Stage 5 (eGFR <15)** | 0 (0.0) | 0 (0.0) | 0 (0.0) |  |

* N=1211

** N=1216

*** N=938

**** N=355

Categorical variables were presented as a percentage and continuous variables were presented as mean and standard deviation.
Abbreviation: CKD, chronic kidney disease; CrCl, Creatinine clearance; DAPT, dual antiplatelet therapy; eGFR, estimated glomerular filtration rate; EHRA, European Heart Rhythm Association; MDRD, modification of diet in renal disease; NOAC, Non-Vitamin K antagonist oral anticoagulants; PT INR, prothrombin time international normalized ratio; SAPT, single antiplatelet therapy; TIA, transient ischemic attack; VKA, Vitamin K antagonist.

**(C) Serum Creatinine ≥1.5 mg/dL**

|  | **Total**  **(n=156)** | **Apixaban dose** | | ***p*-value** |
| --- | --- | --- | --- | --- |
|  |  | **On-label  standard-dose**  **(n=52)** | **Off-label  reduced dose**  **(n=104)** |  |
| **Age, years** | 70.7 ± 7.2 | 68.3 ± 10.6 | 70.5 ± 9.4 | 0.215 |
| **<65** | 26 (16.7) | 10 (19.2) | 16 (15.4) | 0.543 |
| **65–74** | 72 (46.2) | 25 (48.1) | 47 (45.2) | 0.733 |
| **75–79** | 58 (37.2) | 17 (32.7) | 41 (39.4) | 0.412 |
| **Sex (female)** | 20 (12.8) | 2 (3.8) | 18 (17.3) | 0.018 |
| **Bodyweight (kg)** | 75.2 ± 11.6 | 80.1 ± 8.2 | 73.8 ± 9.9 | 0.001 |
| **60–65** | 32 (20.5) | 3 (5.8) | 29 (27.9) | 0.001 |
| **CHA₂DS₂-VASc score** | 3.3 ± 1.2 | 2.6 ± 1.0 | 3.2 ± 1.2 | 0.291 |
| **≥3** | 113 (72.4) | 40 (76.9) | 73 (70.2) | 0.375 |
| **HAS-BLED score^*^** | 2.0 ± 1.0 | 2.3 ± 1.5 | 2.2 ± 1.2 | 0.537 |
| **≥3** | 41 (26.3) | 9 (17.3) | 32 (30.8) | 0.055 |
| **Comorbidities** |  |  |  |  |
| **Hypertension** | 126 (80.8) | 43 (82.7) | 83 (79.8) | 0.667 |
| **Diabetes Mellitus** | 92 (59.0) | 25 (48.1) | 67 (64.4) | 0.050 |
| **Heart failure** | 48 (30.8) | 19 (36.5) | 29 (27.9) | 0.270 |
| **Prior stroke/TIA** | 24 (15.4) | 9 (17.3) | 15 (14.4) | 0.638 |
| **Prior bleeding** | 20 (12.8) | 6 (11.5) | 14 (13.5) | 0.735 |
| **CKD** | 109 (69.9) | 35 (67.3) | 74 (71.2) | 0.622 |
| **Not on dialysis** | 87 (55.8) | 31 (59.6) | 56 (53.8) | 0.145 |
| **On dialysis** | 20 (12.8) | 4 (7.7) | 16 (15.4) | 0.189 |
| **Previous kidney transplantation** | 1 (0.6) | 0 (0.0) | 1 (1.0) | 1.000 |
| **Liver disease** | 6 (3.8) | 1 (1.9) | 5 (4.8) | 0.664 |
| **Malignancy** | 29 (18.6) | 8 (15.4) | 21 (20.2) | 0.467 |
| **Antiplatelet use^**^** | 19 (12.2) | 5 (9.6) | 14 (13.5) | 0.438 |
| **SAPT** | 15 (9.6) | 3 (5.8) | 12 (11.5) | 0.530 |
| **DAPT** | 2 (1.3) | 1 (1.9) | 1 (1.0) | 1.000 |
| **Prior OAC** | 117 (75.0) | 43 (82.7) | 74 (71.2) | 0.373 |
| **Prior VKA** | 7 (4.5) | 3 (5.8) | 4 (3.8) | 1.000 |
| **Prior NOAC** | 110 (70.5) | 40 (76.9) | 70 (67.3) | 0.469 |
| **Apixaban** | 69 (44.2) | 24 (46.2) | 45 (43.3) | 0.504 |
| **Dabigatran** | 4 (2.6) | 2 (3.8) | 2 (1.9) | 0.628 |
| **Edoxaban** | 17 (10.9) | 7 (13.5) | 10 (9.6) | 0.720 |
| **Rivaroxaban** | 20 (12.8) | 7 (13.5) | 13 (12.5) | 0.816 |
| **Atrial fibrillation type** |  |  |  |  |
| **Not determined** | 7 (4.5) | 4 (7.7) | 3 (2.9) | 0.223 |
| **Paroxysmal** | 66 (42.3) | 21 (40.4) | 45 (43.3) | 0.731 |
| **Non paroxysmal** | 83 (53.2) | 27 (51.9) | 56 (53.8) | 0.820 |
| **Persistent** | 67 (42.9) | 22 (42.3) | 45 (43.3) | 1.000 |
| **Long-standing persistent** | 9 (5.8) | 4 (7.7) | 5 (4.8) | 0.710 |
| **Permanent** | 5 (3.2) | 1 (1.9) | 4 (3.8) | 0.660 |
| **EHRA classification** |  |  |  |  |
| **I** | 33 (21.2) | 10 (19.2) | 23 (22.1) | 0.678 |
| **IIa** | 57 (36.5) | 18 (34.6) | 39 (37.5) | 0.724 |
| **IIb** | 23 (14.7) | 8 (15.4) | 15 (14.4) | 0.873 |
| **III** | 4 (2.6) | 1 (1.9) | 3 (2.9) | 1.000 |
| **IV** | 0 (0.0) | 0 (0.0) | 0 (0.0) |  |
| **Unknown** | 39 (25.0) | 15 (28.8) | 24 (23.1) | 0.433 |
| **Lab** |  |  |  |  |
| **Haemoglobin (g/dL) ^***^** | 12.6 ± 2.2 | 13.0 ± 2.1 | 12.2 ± 1.9 | 0.083 |
| **Anemia (men ≤13 g/dL, female ≤12 g/dL)** | 72 | 19 | 53 | 0.229 |
| **Platelet (x10^3^/μl)** | 200.4 ± 74.7 | 203.1 ± 58.8 | 205.7 ± 65.9 | 0.480 |
| **PT INR ^****^** | 1.3 ± 0.4 | 1.4 ± 0.6 | 1.2 ± 0.3 | 0.398 |
| **Creatinine (mg/dL)** | 2.4 ± 1.9 | 3.6 ± 4.1 | 2.9 ± 2.0 | <0001 |
| **CrCl (ml/min)** | 36.1 ± 13.8 | 34.2 ± 15.1 | 31.8 ± 14.2 | <0001 |
| **CrCl <50** | 139 (89.1) | 42 (80.8) | 97 (93.3) | 0.025 |
| **eGFR (MDRD) (mL/min/1.73m^2^)** | 32.9 ± 11.1 | 29.3 ± 13.6 | 29.2 ± 13.0 | <0001 |
| **eGFR (CKD-EPI) (mL/min/1.73m^2^)** | 32.6 ± 11.3 | 29.0 ± 13.9 | 28.9 ± 13.6 | <0001 |
| **Stage 1 (eGFR ≥90)** | 0 (0.0) | 0 (0.0) | 0 (0.0) |  |
| **Stage 2 (60 ≤eGFR <90)** | 0 (0.0) | 0 (0.0) | 0 (0.0) |  |
| **Stage 3a (45 ≤eGFR <60)** | 9 (5.8) | 5 (9.6) | 4 (3.8) | 0.274 |
| **Stage 3b (30 ≤eGFR <45)** | 89 (57.1) | 38 (73.1) | 51 (49.0) | 0.005 |
| **Stage 4 (15 ≤eGFR <30)** | 42 (26.9) | 6 (11.5) | 36 (34.6) | 0.002 |
| **Stage 5 (eGFR <15)** | 15 (9.6) | 3 (5.8) | 12 (11.5) | 0.242 |

* N=147

** N=152

*** N=133

**** N=38

Categorical variables were presented as a percentage and continuous variables were presented as mean and standard deviation.
Abbreviation: CKD, chronic kidney disease; CrCl, Creatinine clearance; DAPT, dual antiplatelet therapy; eGFR, estimated glomerular filtration rate; EHRA, European Heart Rhythm Association; MDRD, modification of diet in renal disease; NOAC, Non-Vitamin K antagonist oral anticoagulants; PT INR, prothrombin time international normalized ratio; SAPT, single antiplatelet therapy; TIA, transient ischemic

**Table S3. Baseline characteristics of the apixaban on-label standard-dose, marginal off-label reduced dose group, and non-marginal off-label reduced dose group.**

|  | **Total**  **(n=2000)** | **Apixaban** | | | ***p*-value^†^** | ***p*-value^‡^** |
| --- | --- | --- | --- | --- | --- | --- |
|  |  | **On-label standard- dose**  **(n=974)** | **Marginal  off-label reduced dose**  **(n=496)** | **Non-marginal  off-label reduced dose**  **(n=530)** |  |  |
| **Single dose reduction criteria** |  |  |  |  |  |  |
| **Age ≥80 years** | 593 (29.7) | 247 (25.4) | 174 (35.1) | 172 (32.5) | <0.001 | 0.374 |
| **Bodyweight ≤60 kg** | 1251 (62.6) | 675 (69.3) | 272 (54.4) | 304 (57.4) | <0.001 | 0.416 |
| **Creatinine ≥1.5 mg/dL** | 156 (7.8) | 52 (5.3) | 50 (10.1) | 54 (10.2) | <0.001 | 0.954 |
| **Age, years** | 74.3 ± 7.9 | 72.7 ± 8.1 | 78.4 ± 4.9 | 73.8 ± 8.1 | <0.001 | <0.001 |
| **<65** | 225 (11.3) | 152 (15.6) | 9 (1.6) | 64 (14.1) | <0.001 | <0.001 |
| **65–74** | 691 (34.5) | 410 (42.1) | 36 (6.3) | 245 (54.0) | <0.001 | <0.001 |
| **75–79** | 491 (24.6) | 165 (16.9) | 278 (56.0) | 48 (9.1) | <0.001 | <0.001 |
| **Sex (female)** | 1115 (55.8) | 551 (56.6) | 308 (53.8) | 256 (56.4) | 0.554 | 0.965 |
| **Bodyweight (kg)** | 60.1 ± 10.0 | 59.8 ± 9.6 | 59.4 ± 9.3 | 61.2 ± 11.0 | 0.064 | 0.056 |
| **≤50 kg** | 253 (12.7) | 107 (11.0) | 76 (15.3) | 70 (13.2) | 0.055 | 0.333 |
| **51–60** | 998 (49.9) | 568 (58.3) | 196 (39.5) | 234 (44.2) | <0.001 | 0.133 |
| **>60** | 749 (37.5) | 299 (30.7) | 224 (45.2) | 226 (42.6) | <0.001 | 0.416 |
| **CHA₂DS₂-VASc score** | 3.4 ± 1.2 | 3.2 ± 1.2 | 3.8 ± 1.1 | 3.2 ± 1.1 | <0.001 | <0.001 |
| **≥3** | 1492 (74.6) | 668 (68.6) | 442 (89.1) | 382 (72.1) | <0.001 | <0.001 |
| **HAS-BLED score ^*^** | 1.6 ± 0.9 | 1.6 ± 0.9 | 1.7 ± 0.8 | 1.6 ± 0.9 | 0.015 | 0.192 |
| **≥3** | 265 (13.7) | 120 (12.6) | 74 (15.6) | 71 (14.0) | 0.293 | 0.495 |
| **Comorbidities** | 1392 (69.9) | 653 (67.0) | 359 (72.4) | 380 (71.7) | 0.052 | 0.808 |
| **Hypertension** | 610 (30.5) | 283 (29.1) | 158 (31.9) | 169 (31.9) | 0.393 | 0.991 |
| **Diabetes Mellitus** | 476 (23.8) | 207 (21.3) | 125 (25.2) | 144 (27.2) | 0.025 | 0.474 |
| **Heart failure** | 212 (10.6) | 128 (13.1) | 45 (9.1) | 39 (7.4) | 0.001 | 0.317 |
| **Prior stroke/TIA** | 143 (7.1) | 54 (5.5) | 44 (8.9) | 45 (8.5) | 0.024 | 0.829 |
| **Prior bleeding** | 218 (10.9) | 66 (6.8) | 79 (15.9) | 73 (13.8) | <0.001 | 0.332 |
| **CKD** | 178 (8.9) | 56 (5.7) | 66 (13.3) | 56 (10.6) | <0.001 | 0.175 |
| **Not on dialysis** | 25 (1.3) | 8 (0.8) | 10 (2.0) | 7 (1.3) | 0.147 | 0.383 |
| **On dialysis** | 14 (0.7) | 2 (0.2) | 3 (0.6) | 9 (1.7) | 0.004 | 0.104 |
| **Previous kidney transplantation** | 77 (3.9) | 35 (3.6) | 22 (4.4) | 20 (3.8) | 0.726 | 0.593 |
| **Liver disease** | 270 (13.5) | 122 (12.5) | 83 (16.7) | 65 (12.3) | 0.052 | 0.042 |
| **Malignancy** | 125 (6.4) | 43 (4.4) | 42 (8.5) | 40 (7.5) | 0.003 | 0.579 |
| **Antiplatelet use^**^** | 106 (5.3) | 36 (3.7) | 38 (7.7) | 32 (6.0) | 0.406 | 0.180 |
| **SAPT** | 6 (0.3) | 3 (0.3) | 2 (0.4) | 1 (0.2) | 0.635 | 0.586 |
| **DAPT** | 1472 (73.6) | 721 (74.0) | 357 (72.0) | 394 (74.3) | 0.494 | 0.798 |
| **Prior OAC** | 54 (2.7) | 23 (2.4) | 9 (1.8) | 22 (4.2) | 0.054 | 0.034 |
| **Prior VKA** | 1418 (70.9) | 698 (71.7) | 348 (70.2) | 372 (70.2) | 0.113 | 0.167 |
| **Prior NOAC** | 851 (42.6) | 447 (45.9) | 200 (40.3) | 204 (38.5) | 0.002 | 0.276 |
| **Apixaban** | 99 (5.0) | 51 (5.2) | 20 (4.0) | 28 (5.3) | 0.600 | 0.394 |
| **Dabigatran** | 319 (16.0) | 134 (13.8) | 85 (17.1) | 100 (18.9) | 0.023 | 0.600 |
| **Edoxaban** | 149 (7.5) | 66 (6.8) | 43 (8.7) | 40 (7.5) | 0.369 | 0.420 |
| **Rivaroxaban** |  |  |  |  |  |  |
| **Atrial fibrillation type** | 83 (4.2) | 32 (3.3) | 21 (4.2) | 30 (5.7) | 0.087 | 0.293 |
| **Not determined** | 1044 (52.2) | 516 (53.0) | 251 (50.6) | 277 (52.3) | 0.690 | 0.595 |
| **Paroxysmal** | 873 (43.7) | 426 (43.7) | 224 (45.2) | 223 (42.1) | 0.607 | 0.319 |
| **Non paroxysmal** | 705 (35.3) | 334 (34.3) | 190 (38.3) | 181 (34.2) | 0.259 | 0.166 |
| **Persistent** | 92 (4.6) | 48 (4.9) | 25 (5.0) | 19 (3.6) | 0.427 | 0.250 |
| **Long-standing persistent** | 72 (3.6) | 43 (4.4) | 8 (1.6) | 21 (4.0) | 0.021 | 0.023 |
| **Permanent** | 4 (0.2) | 1 (0.1) | 1 (0.2) | 2 (0.4) | 0.523 | 0.602 |
| **EHRA classification** |  |  |  |  |  |  |
| **I** | 478 (23.9) | 225 (23.1) | 135 (27.2) | 18 (23.6) | 0.127 | 0.066 |
| **IIa** | 790 (39.5) | 387 (39.7) | 194 (39.1) | 209 (39.4) | 0.973 | 0.916 |
| **IIb** | 244 (12.2) | 104 (10.7) | 59 (11.9) | 81 (15.3) | 0.032 | 0.114 |
| **III** | 57 (2.9) | 20 (2.1) | 12 (2.4) | 25 (4.7) | 0.010 | 0.049 |
| **IV** | 2 (0.1) | 1 (0.1) | 1 (0.2) | 0 (0.0) | 0.742 | 0.483 |
| **Unknown** | 429 (21.4) | 237 (24.3) | 95 (19.2) | 97 (18.3) | 0.009 | 0.727 |
| **Lab** |  |  |  |  |  |  |
| **Haemoglobin (g/dL) ^***^** | 13.0 ± 1.8 | 13.0 ± 1.9 | 12.3 ± 1.8 | 12.7 ± 1.8 | <0.001 | 0.046 |
| **Anemia (men ≤13 g/dL, female ≤12 g/dL)** | 537 (26.9) | 229 (23.5) | 163 (32.9) | 146 (27.5) | <0.001 | 0.022 |
| **Platelet (x10^3^/μl)** | 207.8 ± 69.2 | 209.0 ± 75.1 | 204.4 ± 56.5 | 212.1 ± 76.9 | 0.236 | 0.322 |
| **PT INR ^****^** | 1.2 ± 0.5 | 1.2 ± 0.3 | 1.2 ± 0.4 | 1.2 ± 0.8 | 0.869 | 0.617 |
| **Creatinine (mg/dL)** | 1.0 ± 0.7 | 1.0 ± 0.9 | 1.1 ± 0.9 | 1.1 ± 0.9 | <0.001 | 0.536 |
| **CrCl (ml/min)** | 56.2 ± 17.4 | 59.4 ± 17.3 | 49.3 ± 14.2 | 56.2 ± 21.1 | <0.001 | <0.001 |
| **CrCl <50** | 733 (36.7) | 276 (28.3) | 271 (54.6) | 186 (35.1) | <0.001 | <0.001 |
| **eGFR (MDRD) (mL/min/1.73m^2^)** | 72.4 ± 23.0 | 76.8 ± 23.4 | 68.1 ± 23.4 | 71.8 ± 28.5 | <0.001 | 0.171 |
| **eGFR (CKD-EPI) (mL/min/1.73m^2^)** | 71.0 ± 19.9 | 75.0 ± 18.7 | 65.4 ± 19.9 | 69.4 ± 22.1 | <0.001 | 0.008 |
| **Stage 1 (eGFR ≥90)** | 276 (13.8) | 185 (19.0) | 23 (4.6) | 68 (12.8) | <0.001 | <0.001 |
| **Stage 2 (60 ≤eGFR <90)** | 1123 (56.2) | 578 (59.3) | 268 (54.0) | 277 (52.3) | 0.072 | 0.633 |
| **Stage 3a (45 ≤eGFR <60)** | 344 (17.2) | 137 (14.1) | 121 (24.4) | 86 (16.2) | <0.001 | 0.001 |
| **Stage 3b (30 ≤eGFR <45)** | 156 (7.8) | 55 (5.6) | 43 (8.7) | 58 (10.9) | <0.001 | 0.210 |
| **Stage 4 (15 ≤eGFR <30)** | 42 (2.1) | 6 (0.6) | 21 (4.2) | 15 (2.8) | <0.001 | 0.229 |
| **Stage 5 (eGFR <15)** | 15 (0.8) | 3 (0.3) | 5 (1.0) | 7 (1.3) | 0.063 | 0.634 |

* N= 1934

** N= 1939

*** N= 1532

**** N= 544

† p-value of three groups of on-label standard-dose, marginal off-label reduced dose, and non-marginal off-label reduced dose

‡p-value between marginal off-label reduced dose and non-marginal off-label reduced dose.

Categorical variables were presented as a percentage and continuous variables were presented as mean and standard deviation.
Abbreviation: CKD, chronic kidney disease; CrCl, Creatinine clearance; DAPT, dual antiplatelet therapy; eGFR, estimated glomerular filtration rate; EHRA, European Heart Rhythm Association; MDRD, modification of diet in renal disease; NOAC, Non-Vitamin K antagonist oral anticoagulants; PT INR, prothrombin time international normalized ratio; SAPT, single antiplatelet therapy; TIA, transient ischemic attack; VKA, Vitamin K antagonist.

**Figure S1.** Distribution of subjects in each criterion according to remaining two criteria


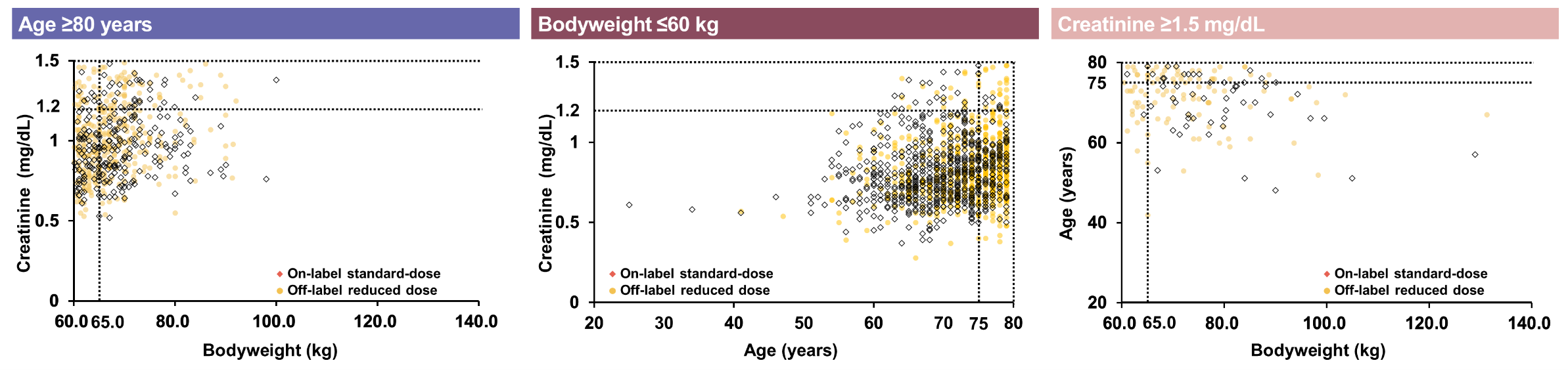

Supplement: Supplementary file 1 [file Table1.docx]
